# Determinants of late neonatal nosocomial infection: a case-control study in Ceará

**DOI:** 10.11606/s1518-8787.2022056003291

**Published:** 2022-05-18

**Authors:** Carmen Sulinete Suliano da Costa Lima, Hermano Alexandre Lima Rocha, David Augusto Batista Sá Araújo, Cláudia Silva

**Affiliations:** I Universidade Fernando Pessoa Faculdade de Ciências e Tecnologia Porto Portugal Universidade Fernando Pessoa. Faculdade de Ciências e Tecnologia. Porto, Portugal; II Harvard S,chool of Public Health Boston MA USA Harvard S,chool of Public Health. Global Health and Population. Boston, MA, USA; III Universidade Federal do Ceará Departamento de Saúde Comunitária Fortaleza CE Brasil Universidade Federal do Ceará. Departamento de Saúde Comunitária. Fortaleza, CE, Brasil; IV Universidade Fernando Pessoa Unidade de Investigação em Energia, Ambiente e Saúde Porto Portugal Universidade Fernando Pessoa. Unidade de Investigação em Energia, Ambiente e Saúde. Porto, Portugal

**Keywords:** Infant, Newborn, Infant, Premature, Cross Infection, epidemiology, Drug Resistance, Multiple, Bacterial, Risk Factors, Case-Control Studies

## Abstract

**OBJECTIVES:**

To assess the determining factors of late healthcare-associated infections (HAIs) and bacterial multiple drug resistance in neonatal intensive care.

**METHODS:**

This is a case-control study, conducted between January 2013 and December 2017, in a neonatal intensive care unit in the state of Ceará, Brazil. Newborns showing late HAIs were considered cases and those without infection, the control. Variables with p-values ≤ 0.05 in our initial bivariate regressive analysis were included in a non-conditional hierarchical logistic model for multivariate analysis. P-values below 0.01 were considered significant.

**RESULTS:**

Of the 1,132 participants, 427 (37.7%) showed late healthcare-associated infections. Of these, 54 (12.6%), positive blood cultures, of which 14.9% contained multidrug-resistant bacteria. Bivariate analysis showed the protective effect of the feminine phenotype (OR = 0.71; 95%CI: 0.56–0.90) and of gestational ages ≥ 34 weeks (OR = 0.48; 95%CI: 0.30–0.75). In earlier-born preterm infants, late infections were 18 times more likely in those with less than 30 week-gestations (OR = 18.61; 95%CI: 9.84–35.22) and four times higher in those weighing less than 1,500 g (OR = 4.18; 95%CI: 3.12–5.61). Mechanical ventilation increased infection odds by more than seven times (OR = 7.14; 95%CI: 5.26–9.09); as did parenteral nutrition (OR = 5.88; 95%CI: 4.54–7.69); central venous catheters (OR = 10.00; 95%CI: 6.66–16.66); the number of catheters used (OR = 3.93; 95%CI: 3.02–5.12); surgery (OR = 4.00; 95%CI: 2.27–7.14); and hospitalization time (OR = 1.06; 95%CI: 1.05–1.07). The association between preterm infants with less than 30 week-gestations (OR = 5.62; 95%CI: 1.83–17.28); mechanical ventilation (OR = 1.84; 95%CI: 1.26–2.68); central venous catheters (OR = 2.48; 95%CI: 1.40–4.37); and hospitalization time (OR = 1.06; 95%CI: 1.05–1.07) remained significant after adjustment. Among deaths, 41 (55.4%) were associated with late infections.

**CONCLUSION:**

Better practices should be adopted in caring for the premature, as well as in the rational use of procedures, to avoid late healthcare-associated infections, preventable deaths, and risks of bacterial multiple drug resistance and environmental contamination.

## INTRODUCTION

Healthcare-associated infections (HAIs) are acquired in health services when infectious agents affect susceptible hosts, causing a disease. These infectious agents can be bacteria, viruses, fungi or parasites present in reservoirs, which could be patients, healthcare providers, visitors or hospital surfaces and inanimate objects^1^. Failures in environmental cleaning, article and clothing processing, and standard precautions cause surfaces or objects to harbor microorganisms which can lead to cross-contamination^2 , 3^. The term hospital infection has been gradually replaced by late HAIs, as it is more comprehensive and includes infections related to hospital care, care failures, prevention, diagnosis, and treatment^4^.

Neonatology defines hospital-derived late HAIs as infections whose diagnostic evidence occurs after hospitalized newborns’ first 48 hours of life^4^. These patients are more susceptible to infections, especially if premature and with very low weights since their immune systems are still in development and have inefficient mucous and cutaneous barriers, in addition to their greater exposure to objects used in care, which may be contaminated^3 , 5^, and to many high-risk therapeutic interventions, such as the use of broad-spectrum invasive and antimicrobial devices which influence colonization processes^6 , 7^.

In recent decades, the survival of premature neonates and patients with malformations previously considered incompatible with life increased. However, prolonging their hospitalization in neonatal intensive care units (NICUs) increased complications, such as late HAIs, which became a limiting factor for their survival^4 , 8^. According to a report released in 2016 by the United Nations Children’s Fund, infections (sepsis, meningitis, and pneumonia) are responsible for about 21% of neonatal deaths worldwide, representing a serious public health problem^9^. Infections relate to birth weight, especially in low birth weight newborns or preterm infants, and are associated with invasive procedures and long hospital stays^10^; the use of a central venous catheter (CVC); and period of mechanical ventilation^4 , 11^, causing mainly pneumonia and primary bloodstream infections; the latter if hospitalized patients receive catheters^4^.

In Brazil, 60% of infant mortality occurs in the neonatal period^4^, and its main cause are infections^4 , 6 , 12^. Primary bloodstream infections associated with CVC are the main type of infection in Brazilian NICUs^4^. In a study of deaths in NICUs in the state of Ceará, Brazil, the main causes of death were HAIs (44%), followed by diseases related to prematurity, respiratory diseases, and asphyxia^13^. A Brazilian retrospective cohort study, conducted in Rio de Janeiro between 2008 and 2012, found an association between invasive procedures and infection, but only included 49 very low birth weight newborns^14^. A prospective cohort study, conducted in 2013 in a hospital in Porto Alegre, in the state of Rio Grande do Sul, analyzed 30 preterm infants weighing less than 1,500 g and neonatal infections, describing the clinical characteristics and use of technologies in that NICU, but without statistically associating risk factors^15^. Another descriptive study, conducted in 2010 in a NICU in the state of Santa Catarina, Brazil found that 45.8% of hospitalized patients had late HAIs, but failed to estimate measures of association^16^. Due to their scarcity, studies are needed in both Ceará and Northeastern Brazil which evaluate risk factors for late HAIs, assess this epidemiological reality, improve NICU practices and, consequently, reduce neonatal morbidity and mortality.

NICUs often face the empirical treatment of late HAIs. The inadequate use of several antimicrobials, in turn, is the main cause of bacterial resistance to drugs, which may cause the emergence of multidrug-resistant bacteria^17^, which would risk their eventual endemicity^18^. Since the late 1980s, Latin American NICUs have witnessed an increase in bacteria highly resistant to common antibiotics, mainly due to prolonged patient hospitalization, insertion of invasive devices, and non-compliance with safety and isolation standards^11 , 19^.

This study aimed to evaluate the risk factors associated with late HAIs to assess bacterial flora and its response to antimicrobials, pinpointing the presence or not of multidrug-resistant bacteria to propose strategies to prevent health problems, and reduce the occurrence of preventable deaths and the spread of harmful bacteria in NICUs. Late HAIs are a threat to neonates and the entire in-hospital environment, and constitute a risk of intrapersonal external and environmental contamination.

## METHODS

This is a case-control study, conducted between January 2013 and December 2017, on the characteristics of newborns diagnosed with late HAIs in a NICU of a public reference hospital in the state of Ceará, Brazil.

The *Hospital Geral Waldemar Alcântara* (Waldemar Alcântara General Hospital) is a secondary care unit within the public health network and the first public hospital in Northern and Northeastern Brazil to receive a level 2 hospital accreditation from the *Organização Nacional de Acreditação* (National Accreditation Organization). It is a support hospital for the tertiary care network of the state of Ceará, exclusively serving clients of the Unified Health System. It offers the population 336 beds, distributed in medical, surgical, and pediatric units, special care units, adult intensive care units, NICUs, pediatric intensive care units, and medium risk nurseries, as well as outpatient and home care.

Our studied population consisted of all the newborns hospitalized in the NICU aged up to incomplete 28 days, whether they were included as cases (with late HAIs) or controls (without late HAIs). All patients admitted for the first time to the NICU with 28 complete days of life or more and those with hospital stays shorter than 48 hours were excluded from our study. All patients who were affected by late HAIs during the study period were considered cases, according to the criteria standardized and notified by the national Hospital Infection Control Service. Those unaffected were considered the control. Data were collected from the medical records of hospitalized patients who met the inclusion criteria, via a checklist elaborated by the researcher.

Neonatology defines hospital-derived late HAIs as infections whose diagnostic evidence occurs after hospitalized newborns’ first 48 hours of life^4^. The dependent variable in this study was whether NICU patients showed late HAIs.

The independent variables collected were grouped into two hierarchical strata:

- newborns’ clinical data (gestational age, in weeks by the Capurro or New-Ballard Method. Those born before 37 gestational weeks were considered premature. Their ages were stratified in: up to 29 weeks and six days, between 30 and 33 weeks and six days, between 34 and 36 weeks and six days, and greater than or equal to 37 weeks); sex (male or female); birth weight (in grams. Babies born with a weight below 2,500 g were considered with low birth weight, stratified in: less than 1,500 g, between 1,500 g and 2,499 g, and greater than or equal to 2,500 g); and the fifth-minute Apgar index as an indicator of asphyxia (< 7 or ≥ 7 score);
- hospital care data [use of mechanical ventilation (yes or no); ventilation period (in days); use of CVC (yes or no); number of CVCs used; CVC use period (in days); parenteral nutrition (yes or no); parenteral nutrition period (in days); surgery (yes or no); and total hospitalization period (in days)] ( Figure ).
  **Figure:**
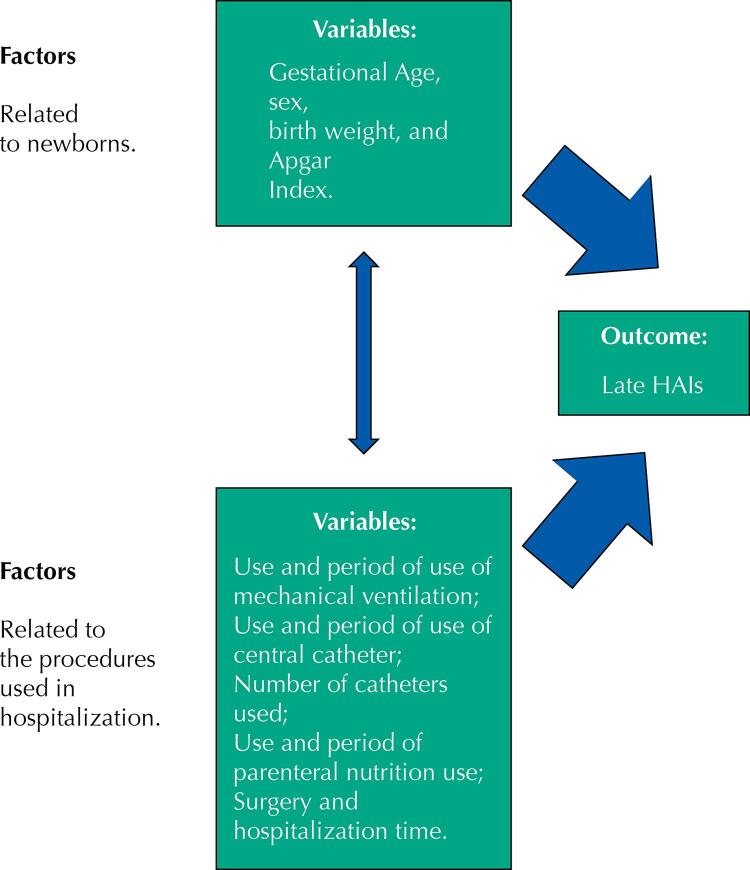
Hierarchical conceptual structure for the study of late HAIs in a Neonatal Intensive Care Unit.

The type of isolated microorganism (gram-negative, gram-positive, and anaerobic bacteria; or fungi) and the presence or not of multidrug-resistant bacteria were included; in addition to the occurrence of deaths among cases.

Initially, a univariate descriptive statistical analysis was performed for medians (continuous variables) and proportions (categorical variables). To estimate the association between dependent and independent variables, bivariate analyses were performed to evaluate the significance of the associations (Chi-square and Kruskal-Wallis tests). Odds Ratio (OR) were used as the association measure. Dependent (late HAIs: present or absent) and independent variables were submitted to bivariate analysis to estimate their OR, and their respective 95% confidence intervals (95%CI) were assessed via logistic regression models. Subsequently, the variables showing p ≤ 0.05 were included in the logistic model for a hierarchical multivariate analysis, according to our theoretical model, and considered as significant if their p-values ≤ 0.01, in the final model, after backward stepwise selection. Data were stored and analyzed by SAS 9.4, SAS Inc, and SPSS (Statistical Package for the Social Sciences) Statistics^®^ 26.0, IBM Inc.

Our study was approved by the Ethics Committee of the hospital (Protocol 61/2017) and Plataforma Brasil (Opinion 160/2011).

## RESULTS

We found 1,132 eligible records, of which we excluded 102 patients hospitalized during our study due to incomplete medical records in relation to variables of care and 22 patients since they died in less than 48 hours of hospitalization. Among the 74 deaths that remained in the analysis (6.5% of the records), 41 (55.4%) were due to HAIs, occurring between three and 114 days of life.

Regarding outcomes, 427 records (37.7%) showed late HAIs, of which most were males (59.9%). Gestational ages ranged from 22 to 42 weeks. In total, 62.5% of babies were premature whose gestational ages were predominantly greater than or equal to 30 weeks and less than 34 weeks (37.5%). Birth weight ranged from 415 g to 6,200 g, and 60.7% were underweight (less than 2,500 g). Of the valid scores of the fifth-minute Apgar index, 27% showed a value lower than seven.

In the bivariate analysis of newborns’ clinical variables, we found a protective effect against late HAIs in female infants (OR = 0.71; 95%CI: 0.56–0.90) and in premature newborns with gestational ages equal to 34 weeks or above and below 37 weeks (OR = 0.48; 95%CI: 0.30–0.75). The more premature, the greater the association with late HAIs; almost twice as high in preterm infants with gestational ages equal to 30 weeks or above and below 34 weeks (OR = 1.93; 95%CI: 1.45–2.57), and about 18 times higher in preterm infants with gestational ages below 30 weeks (OR = 18.61; 95%CI: 9.84–35.22). Newborns with birth weights below 1,500 g showed about four times as much association with late HAIs (OR = 4.18; 95%CI: 3.12–5.61), and children under 2,500 g showed a risk about twice as high of late HAIs (OR = 2.27; 95%CI: 1.75–2.94). Asphyxia was associated with a risk about three times higher of late HAIs (OR = 2.94; 95%CI: 2.17–3.84) ( Table 1 ). We observed that all variables show statistically significant associations with late HAIs (p < 0.0001), except sex ( Table 1 ) and parenteral nutrition period ( Table 2 ).

**Table 1:** Distribution of cases and controls, with respective odds ratios for the variables related to newborns’ clinical data. Ceará, Brazil, 2013 to 2017.

	Late HAIs		OR (95%CI)	p
	Present (n = 427) Cases	Absent (n = 705) Control		
Sex	n (%)	n (%)		0.0065^b^
Feminine	193 (45.2%)	261 (37.0%)	0.71 (0.56–0.90)	
Masculine	234 (54.8%)	444 (63.0%)	1.0	
Gestational age classes	n (%)	n (%)		< 0.0001^b^
≥ 37 weeks	122 (28.6%)	303 (43.0%)	1.0	
34 to 36 weeks	29 (6.8%)	151 (21.4%)	0.48 (0.30–0.75)	0.0012^a^
30 to 33 weeks	186 (43.6%)	239 (33.9%)	1.93 (1.45–2.57)	< 0.0001^a^
< 30 weeks	90 (21.1%)	12 (1.7%)	18.61 (9.84–35.22)	< 0.0001^a^
Birth weight (g)				< 0.0001^a^
n	427	705		
Median (min-max)	1,405 (415–5,085)	2,370 (550–6,200)		
Birth weight classes	n (%)	n (%)		< 0.0001^b^
> 2,500g	118 (27.6%)	327 (46.4%)	1.0	
Between 1,500g and 2,499g	78 (18.3%)	225 (31.9%)	0.96 (0.69–1.34)	0.8131^a^
< 1,500g	231 (54.1%)	153 (21.7%)	4.18 (3.12–5.61)	< 0.0001^a^
Underweight	n (%)	n (%)		< 0.0001^b^
> 2,500g	118 (27.6%)	327 (46.4%)	1.0	
< 2,500g	309 (72.4%)	378 (53.6%)	2.27 (1.75–2.94)	< 0.0001^a^
Fifth-minute Apgar < 8				< 0.0001^a^
n	385	652		
Median (min-max)	7.0 (0.0–10.0)	8.0 (1.0–10.0)		
Asphyxia	n (%)	n (%)		< 0.0001^b^
Omitted values	42	53		
Apgar ≥ 7	229 (59.5%)	528 (81.0%)	1.0	
Apgar < 7	156 (40.5%)	124 (19.0%)	2.94 (2.17–3.84)	< 0.0001^a^

^a ^Kruskal-Wallis p-value.

^b ^Chi-square p-value.

**Table 2:** Distribution of cases and controls, with respective odds ratios for the variables related to newborns’ clinical data. Ceará, Brazil, 2013 to 2017.

	Late HAIs		OR (95%CI)	p
	Present (n = 427) Cases	Absent (n = 705) Control		
Use of mechanical ventilation	n (%)	n (%)		< 0.0001^b^
No	76 (17.8%)	426 (60.4%)	1.0	
Yes	351 (82.2%)	279 (39.6%)	7.14 (5.26–9.09)	< 0.0001^a^
Ventilation period (days)			1.15 (1.11–1.20)	< 0.0001^a^
n	351	279		
Median (min-max)	9 (1–88)	4 (1–45)		
Use of parenteral nutrition	n (%)	n (%)		< 0.0001^b^
No	117 (27.4%)	484 (68.7%)	1.0	
Yes	310 (72.6%)	221 (31.3%)	5.88 (4.54–7.69)	< 0.0001^a^
Parenteral nutrition time (days)			1.08 (1.05–1.12)	0.0008^a^
n	310	221		
Median (min-max)	6 (1–59)	6 (1–27)		
Use of central catheter	n (%)	n (%)		< 0.0001^b^
No	21 (4.9%)	246 (34.9%)	1.0	
Yes	406 (95.1%)	459 (65.1%)	10.00 (6.66–16.66)	< 0.0001^a^
Central catheter period (days)			1.09 (1.07–1.11)	< 0.0001^a^
n	406	459		
Median (min-max)	10 (1–93)	7 (1–34)		
Number of catheters			3.93 (3.02–5.12)	< 0.0001^a^
n	406	459		
Median (min-max)	2 (1–5)	1 (1–3)		
Surgery	n (%)	n (%)		< 0.0001^b^
No	386 (90.4%)	687 (97.4%)	1.0	
Yes	41 (9.6%)	18 (2.6%)	4.00 (2.27–7.14)	< 0.0001^a^
Hospitalization period (days)			1.06 (1.05–1.07)	< 0.0001^a^
n	427	705		
Median (min-max)	50 (3–198)	14 (2–100)		
Death	n (%)	n (%)		< 0.0001^b^
No	368 (86.2%)	690 (97.9%)	1.0	
Yes	59 (13.8%)	15 (2.1%)	7.14 (4.16–12.50)	< 0.0001^a^

^a ^Kruskal-Wallis p-value.

^b ^Chi-square p-value.

By analyzing clinical procedures, we found that 55.7% of patients received mechanical ventilation for a period ranging from one to 88 days and about half remained intubated for up to five days; 46.9%, parenteral nutrition; and 76.4%, CVC. Most cases (62.2%) received one catheter and 31.3%, two. We also found 59 surgery records (5.2%). Total hospitalization time (including the period at an intermediate risk unit) was up to 21 days for 50% of newborns, ranging from two to 198 days ( Table 2 ).

By assessing these associations, we found that mechanical ventilation increased the risk of late HAIs by about seven times (OR = 7.14; 95%CI: 5.26–9.09); each day of mechanical ventilation increased the chances of infection by 15% (OR = 1.15; 95%CI: 1.11–1.20); whereas parenteral nutrition increased these chances sixfold (OR = 5.88; 95%CI: 4.54–7.69); as did parenteral nutrition period (in days) (OR = 1.08; 95%CI: 1.05–1.12). Moreover, CVCs increased the chance of late HAIs tenfold (OR = 10.00; 95%CI: 6.66–16.66), with each day with catheters increasing this chance by 9% (OR = 1.09; 95%CI: 1.07–1.11). This association also showed a gradient, it increased with the number of CVCs used (OR = 3.93; 95%CI: 3.02–5.12). Surgery was associated with a fourfold higher chance of HAIs (OR = 4.00; 95%CI: 2.27–7.14) and each additional day of hospitalization increased the chances of late HAIs by 6% (OR = 1.06; 95%CI: 1.05–1.07) ( Table 2 ).

Of the cases analyzed, 164 had two or more infectious episodes, thus distributed: 50 patients had two late HAIs; 20, three; 10, four; six, five; two, six; and one, seven late HAIs. Among those reported with late HAIs, 54 (12.6%) showed a positive blood culture record for bacterial infections (n = 47); seven (14.9%), for multidrug-resistant bacteria; and seven (13%), for fungi. Table 3 describes the bacteria and/or fungi found.

**Table 3:** Results of positive blood cultures in late HAIs in the studied NICU. Ceará, Brazil, 2013 to 2017.

Blood culture	Absolute frequency (n)	Relative frequency (%)
Gram-negative bacteria	26	48.1
Gram-positive bacteria	21	38.9
Fungi	7	13.0
Total	54	100.0

After adjustment in the final model, the hierarchical multivariate analysis of outcomes showed significant associations, such as gestational age lower than 30 weeks (OR = 5.62; 95%CI: 1.83–17.28), mechanical ventilation (OR = 1.84; 95%CI: 1.26–2.68), CVC (OR = 2.48; 95%CI: 1.40–4.37), and hospitalization time (OR = 1.06; 95%CI: 1.05–1.07) ( Table 4 ).

**Table 4:** Final model, according to clinical variables and procedures used during hospitalization, with respective odds ratios and 95%CI. Ceará, Brazil, 2013 to 2017.

	Odds ratio (95%CI)	p
Premature < 30 weeks	5.62 (1.83–17.28)	0.003^a^
Use of mechanical ventilation	1.84 (1.26–2.68)	0.001^b^
Use of central catheter	2.48 (1.40–4.37)	0.002^b^
Total length of stay	1.06 (1.05–1.07)	< 0.0001^a^

^a ^Covariate Wald p-value.

^b ^Type 3 Wald p-value.

## DISCUSSION

In this study, we observed a 37.7% occurrence of late HAIs. We also found that chances of infection increase according to how early babies were born, the need for mechanical ventilation and CVCs, and hospitalization periods.

The prevalence of late HAIs was similar to other studies: Egypt, 38.5%^20^; Brazil, 34%^6^; and India, 31%^21^; but much higher than in Taiwan, 11.4%^22^ and in a European multicenter study, 10.7%^23^. This outcome was associated with prematurity among children with gestational ages under 30 weeks, use of mechanical ventilation, CVC, and hospitalization time.

The predominant infectious agents in this study were gram-negative (48.1%) and gram-positive bacteria (38.9%), and fungi (13%); a predominance also observed in an Indian study, which found gram-negative (61%) and gram-positive bacteria (39%)^24^; in an Iranian study, which observed gram-negative (40%) and gram-positive bacteria (20.7%); and fungi (6.7%)^25^; and in an Jordanian study, which reported gram-negative (62%) and gram-positive bacteria (31%), fungi (7%)^26^, and high bacterial resistance (around 39%)^27^; higher than in this study (14.9%).

Our bivariate analysis showed a significant association between HAIs and gestational age in preterm infants, in which children with gestational ages below 30 weeks had an 18-fold greater chance of infection, a value which remained in the final model after adjustments with other variables. Newborns are already naturally susceptible to infections due to the immaturity of their immune defenses^3^. In the presence of other factors, such as prematurity and/or low weight, complication risks increase, as does the need for more invasive procedures and longer hospitalization periods. Due to alveolar surfactant deficiency^28^, preterm infants often show pulmonary immaturity, characterized as respiratory distress syndrome (also called hyaline membrane disease - RDS). Due to RDS, mechanical ventilation is a common procedure to assist lungs in maintaining gas exchange^29^. Regardless of weight, the earlier the babies are born, the greater the RDS severity and the need for invasive procedures, which increase the risk of HAIs. As there is often a direct relationship between lower weight and prematurity, statistical analyses may confound these variables.

In this study, late HAIs was associated with CVC and mechanical ventilation, which also occurred in other studies, although with higher values: CVC (OR = 4.32; 95%CI: 1.95–9.56) and mechanical ventilation (OR = 3.42; 95%CI: 2.17–5.41)^6^. A cross-sectional study, conducted between 2013 and 2015 in Rio Grande do Sul, found that mechanical ventilation and surgery showed a statistically significant association with HAIs in neonates with positive blood cultures (47.97% of those infected)^10^. Another study, conducted from 2001 to 2005 in Londrina, Paraná State, observed a relevant association with intubation and use of CVC^21^.

A meta-analysis of 22 studies (with 2,270 infected and 21,605 healthy newborns), published in 2019^30^, associated as risks for late HAIs: weight < 2,500 g (RR = 3.44; 95%CI: 2.31–5.11), prematurity (RR = 3.85; 95%CI: 1.87–7.92), mechanical ventilation (RR = 3.16; 95%CI: 2.21–4.50), and asphyxia (RR = 1.68; 95%CI: 1.04–2.71). This study also associated HAIs with prematurity and mechanical ventilation. An Indian study found an association with prematurity (OR = 3.05; 95%CI: 1.94–9.88), CVC (OR = 15.11; 95%CI: 3.40–67.01), mechanical ventilation (OR = 8.94; 95%CI: 1.32–60.31), and NICU stays greater than 10 days (OR = 4.09; 95%CI: 1.05–16.70)^25^, as did this study, although with lower values. The use of intravascular catheters has become, in recent years, indispensable in intensive care since it is a high-output vascular access^18^, in addition to the difficulty of peripheral vascular access in newborns. The disadvantage of central accesses is the increased risk of local or systemic infection, which may cause bloodstream infections associated with CVC^18 ,31^. As newborns often require more than one central access, care increases the risk of HAIs^18^. As newborns require longer hospital stays and more invasive procedures, such as central catheters and mechanical ventilation, for example, care also increases the risk of HAIs.

Though the results found in this study are consistent with the national and international literature, there are noteworthy limitations. The retrospective use of secondary data can lead to incomplete registers in the medical records, especially in relation to newborns’ data and the time of use of invasive procedures. This was circumvented by reviewing their entire hospitalization via live birth records, inter-hospital transfer reports, and the daily evolution of the entire multidisciplinary team.

We found that late HAIs are still very frequent in the studied NICU, that preterm infants are still a very vulnerable group to infection risks, and that invasive procedures, essential for most critically ill neonates, should be carefully prescribed.

## CONCLUSIONS

We conclude that, although it is impossible to avoid invasive procedures in critical neonates, insertion, installation, and maintenance of these devices should be rigorously reviewed with hospital infection control and care safety committees, following updated and vigilant protocols. The judicious use of antimicrobials, administered to prevent the emergence of multidrug-resistant bacteria is also a priority action. Regarding prematurity, the best strategy is to prevent premature deliveries since the intrauterine medium, with adequate prenatal follow-up, is most often the safest measure to maintain fetuses in the womb for as long as possible, until near or up to the gestational age of term.
