# Supplementary material for: Determinants of late neonatal nosocomial infection: a case-control study in Ceará
Source: Rev Saude Publica. 2022 May 18;56:40. doi: 10.11606/s1518-8787.2022056003291 (PMC9126576; doi:10.11606/s1518-8787.2022056003291)
Supplement: Translation

# Translation: Determinantes de infecção nosocomial tardia neonatal: estudo de caso-controle no Ceará

**Authors:** Carmen Sulinete Suliano da Costa Lima, Hermano Alexandre Lima Rocha, David Augusto Batista Sá Araújo, Cláudia Silva

**Affiliations:** I Portugal Universidade Fernando Pessoa. Faculdade de Ciências e Tecnologia. Porto, Portugal; II USA Harvard School of Public Health. Global Health and Population. Boston, MA, USA; III Brasil Universidade Federal do Ceará. Departamento de Saúde Comunitária. Fortaleza, CE, Brasil; IV Portugal Universidade Fernando Pessoa. Unidade de Investigação em Energia, Ambiente e Saúde. Porto, Portugal

**Keywords:** Recém-Nascido, Recém-Nascido Prematuro, Infecção Hospitalar, epidemiologia, Farmacorresistência Bacteriana Múltipla, Fatores de Risco, Estudos de Casos e Controles

## Abstract

**OBJETIVO:**

Identificar os fatores determinantes para infecções relacionadas à assistência à saúde (IRAS) tardias em terapia intensiva neonatal e multirresistência bacteriana.

**MÉTODOS:**

Estudo caso-controle em unidade de terapia intensiva neonatal do Estado do Ceará, entre janeiro de 2013 e dezembro de 2017. Considerado caso o recém-nascido notificado como IRAS tardia e, controle, aquele sem infecção. Variáveis com valor de p ≤ 0,05 em análise regressiva bivariada inicial foram incluídas em modelo logístico hierarquizado não condicional para análise multivariada. Valores de p menores que 0,01 foram considerados significativos.

**RESULTADOS:**

Dos 1.132 participantes, 427 (37,7%) tiveram infecções tardias relacionadas a assistência à saúde, com 54 (12,6%) hemoculturas positivas e 14,9% dessas foram bactérias multirresistentes. Na análise bivariada observou-se efeito protetor do sexo feminino (OR = 0,71; IC95% 0,56–0,90) e recém-nascido ≥ 34 semanas (OR = 0,48; IC95% 0,30–0,75). Nos mais prematuros, as infecções tardias tiveram chance dezoito vezes maior em menores do que 30 semanas (OR = 18,61; IC95% 9,84–35,22); e nos menores de 1.500g, quatro vezes maior (OR = 4,18; IC95% 3,12–5,61). O uso de ventilação mecânica aumentou em mais de sete vezes a chance (OR = 7,14; IC95% 5,26–9,09); o mesmo aconteceu com o recurso da nutrição parenteral (OR = 5,88; IC95% 4,54–7,69), com o cateter venoso central (OR = 10,00; IC95% 6,66–16,66); o número de cateteres utilizado (OR = 3,93; IC95% 3,02–5,12); a realização de cirurgia (OR = 4,00; IC95% 2,27–7,14) e o tempo de internamento (OR = 1,06; IC95% 1,05–1,07). Permaneceram significativos após ajuste: prematuro menor do que 30 semanas (OR = 5,62; IC95% 1,83–17,28); uso de ventilação (OR = 1,84; IC95% 1,26–2,68); uso de cateter venoso central (OR = 2,48; IC95% 1,40–4,37) e tempo de internamento (OR = 1,06; IC95% 1,05–1,07). Dentre os óbitos, 41 (55,4%) foram associados às infecções tardias.

**CONCLUSÃO:**

Melhores práticas devem ser adotadas no cuidado da prematuridade e o uso racional de procedimentos, para evitar infecções tardias relacionadas a assistência à saúde, óbitos preveníveis e riscos de multirresistência bacteriana e contaminação ambiental.

## INTRODUÇÃO

As infecções relacionadas à assistência à saúde (IRAS) são adquiridas em serviços de saúde, um agente infeccioso acomete um hospedeiro suscetível, causando uma doença. Esse agente infeccioso pode ser uma bactéria, um vírus, fungo ou parasita, presente em um reservatório que pode ser um paciente, profissional de saúde, visitantes ou superfícies hospitalares e objetos inanimados^1^. As superfícies ou os objetos abrigam microrganismos quando ocorre falhas na limpeza ambiental, no processamento de artigos e roupas e no uso às precauções-padrão, podendo levar à contaminação cruzada^2 , 3^. O termo infecção hospitalar vem sendo substituído gradualmente por IRAS tardia, pois é mais abrangente e inclui as infecções relacionadas aos cuidados hospitalares, às falhas do cuidado, à prevenção, ao diagnóstico e ao tratamento^4^.

IRAS tardia de origem hospitalar em neonatologia é definida como a infecção em que a evidência diagnóstica ocorre após as primeiras 48 horas de vida do recém-nascido internado^4^. O recém-nascido é mais susceptível às infecções, principalmente os prematuros e de muito baixo peso, pois seu sistema imune ainda está em desenvolvimento, possui barreiras mucosas e cutâneas ineficientes, além de estarem mais expostos a objetos utilizados na assistência que podem estar contaminados^3 , 5^e a muitas intervenções terapêuticas de alto risco, como uso de dispositivos invasivos e antimicrobianos de amplo espectro, que influenciam no seu processo de colonização^6 , 7^.

Nas últimas décadas, a sobrevida de neonatos prematuros e de portadores de malformações antes consideradas incompatíveis com a vida aumentou, porém, com o prolongamento do tempo de internação em unidade de terapia intensiva neonatal (UTIN), aumentaram as complicações, entre elas, as IRAS tardias, que passaram a ser um dos fatores limitantes para a sobrevida^4 , 8^. A infecção (sepse, meningite e pneumonia) é responsável por cerca de 21% dos óbitos no período neonatal no mundo, segundo relatório divulgado em 2016 pelo Fundo das Nações Unidas para a Infância, representando um grave problema de saúde pública^9^. A infecção está relacionada ao peso no nascimento, principalmente em recém-nascidos de baixo peso ou em prematuros, e é associada ao uso de procedimentos invasivos e a longa permanência hospitalar^10^; a utilização de cateter venoso central (CVC) e com o tempo de ventilação mecânica^4 , 11^, ocasionando principalmente pneumonia e infecção primária da corrente sanguínea, causada por cateter nos pacientes internados^4^.

No Brasil, 60% da mortalidade infantil ocorre no período neonatal^4^, sendo a infecção uma das principais causas^4 , 6 , 12^. A infecção primária da corrente sanguínea associada ao CVC é a principal infecção em UTIN no Brasil^4^. Em estudo de óbitos em UTIN no Ceará, as principais causas de óbito foram as IRAS (44%), seguidas por afecções relacionadas à prematuridade, afecções respiratórias e asfixia^13^. Em pesquisa brasileira de coorte retrospectivo, realizada no Rio de Janeiro, entre 2008 e 2012, observou-se a associação entre uso de procedimentos invasivos e infecção, porém foram incluídos no trabalho somente 49 recém-nascidos de muito baixo peso^14^. Outra investigação de coorte prospectivo, realizada em um hospital de Porto Alegre, durante o ano de 2013, analisou 30 prematuros com peso inferior a 1.500g e infecção neonatal, descrevendo as características clínicas e uso de tecnologias em UTIN, porém sem avaliar associação estatística dos fatores de risco^15^, e em outro estudo descritivo em uma UTIN de Santa Catarina, no ano de 2010, observou-se que 45,8% dos internados apresentaram IRAS tardia, porém também não foram calculadas medidas de associação^16^. São necessários estudos no Nordeste e no Ceará, que estimem fatores de risco para IRAS tardia, já que existem poucos estudos do tipo, para conhecer a realidade epidemiológica e melhorar as práticas em UTIN e, consequentemente, diminuir a morbimortalidade neonatal.

Em UTIN, o tratamento empírico de IRAS tardia é muito frequente e a utilização inadequada de vários antimicrobianos, por sua vez, é a principal causa de resistência bacteriana a fármacos, podendo ocasionar o aparecimento de bactérias multirresistentes^17^, que podem eventualmente tornar-se endêmicas^18^. Desde o final da década de 1980, na América Latina, há um aumento de bactérias com alta resistência a antibióticos usuais também em UTIN, devido principalmente à internação prolongada, inserção de dispositivos invasivos e ao descumprimento de normas de isolamento e de segurança^11 , 19^.

No presente estudo pretendeu-se avaliar os fatores de risco associados às IRAS tardias, conhecer a flora bacteriana e sua resposta aos antimicrobianos, identificando a presença ou não de bactérias multirresistentes, com a finalidade de propor estratégias de prevenção de agravos à saúde, diminuir a ocorrência de óbitos evitáveis e de propagação de bactérias nocivas na UTIN. As IRAS tardias representam um risco ao neonato e a todo o ambiente intra-hospitalar, além do risco de contaminação externa intrapessoal e ao meio ambiente.

## MÉTODOS

Trata-se de um estudo do tipo caso-controle sobre as características dos recém-nascidos diagnosticados com IRAS tardia em UTIN de um hospital público de referência no Estado do Ceará, no período entre janeiro de 2013 e dezembro de 2017.

O Hospital Geral Waldemar Alcântara é uma unidade de atenção secundária dentro da rede pública de saúde, o primeiro hospital público da região Norte e Nordeste do Brasil a receber o título de acreditação hospitalar nível 2, pela Organização Nacional de Acreditação. Trata-se de um hospital de apoio para a rede terciária de assistência do Estado do Ceará, atendendo exclusivamente à clientela do Sistema Único de Saúde. Oferece à população 336 leitos, distribuídos nas clínicas médicas, cirúrgica, pediátrica, unidade de cuidados especiais, unidade de terapia intensiva de adultos, UTIN, unidade de terapia intensiva pediátrica e berçário médio risco, além de assistência ambulatorial e domiciliar.

A população do estudo foi constituída por todos os recém-nascidos com internamento na UTIN e até 28 dias de vida incompletos, tanto casos (com IRAS tardia) como controles (sem IRAS tardia). Foram excluídos todos os pacientes admitidos pela primeira vez na UTIN com 28 dias de vida ou mais e aqueles com permanência hospitalar menor que 48 horas. Foram considerados casos todos os pacientes que foram acometidos por IRAS tardia no período em estudo, segundo os critérios padronizados e notificados pelo Serviço de Controle de Infecção Hospitalar, e, controles, os que não evoluíram com infecção. Os dados foram coletados de documentos dos prontuários dos pacientes internados que preencheram os critérios de inclusão, com o uso de uma lista de verificação elaborada pela pesquisadora.

IRAS tardia de origem hospitalar foi definida como a infecção que a evidência diagnóstica (clínica/laboratorial/microbiológica) ocorra após as primeiras 48 horas de vida^4^. A variável dependente neste estudo foi a existência de IRAS tardia em UTIN ou não.

As demais variáveis (independentes) recolhidas foram agrupadas em dois estratos hierárquicos:

- dados clínicos do recém-nascido (idade gestacional, em semanas pelo Método de Capurro ou New-Ballard, considerados prematuros os nascidos com menos de 37 semanas e estratificadas as idades gestacionais em: até 29 semanas e seis dias, entre 30 semanas e 33 semanas e seis dias, entre 34 semanas e 36 semanas e seis dias e maior ou igual a 37 semanas); sexo (masculino ou feminino); peso de nascimento (em gramas; considerados de baixo peso os bebês nascidos com menos de 2.500g e estratificados os pesos em: menor do que 1.500g, entre 1.500g e 2.499g, e maior ou igual a 2.500g); e índice de Apgar do 5º minuto como um indicador de asfixia (escore < 7 ou ≥ 7);
- dados da assistência hospitalar [uso de ventilação mecânica (sim ou não); tempo de ventilação (em dias); uso de CVC (sim ou não); número de CVC utilizados; tempo de uso de CVC (em dias); uso de nutrição parenteral (sim ou não); tempo de nutrição parenteral (em dias); cirurgia (sim ou não) e tempo total de internamento (em dias)] ( Figura ).
  **Figura:** Estrutura conceitual hierarquizada para estudo de IRAS tardia em unidade de terapia intensiva neonatal.

Foi incluído o tipo de microrganismo isolado (bactéria Gram negativa, bactéria Gram positiva ou anaeróbia; ou fungo) e a presença ou não de bactéria multirresistente; além da ocorrência ou não de óbitos entre os casos.

Inicialmente, realizou-se a análise estatística descritiva univariada para medianas (variáveis contínuas) e proporções (variáveis categóricas). Para estimar a associação entre a variável dependente e as variáveis independentes, foram realizadas análises bivariadas para testar significância das associações (teste Qui-quadrado e Kruskal-Wallis). A medida de associação utilizada foi *odds ratio* (OR). A variável dependente (IRAS tardia: presente ou ausente) e as variáveis independentes foram submetidas à análise bivariada para as estimativas de OR e respectivos intervalos de confiança de 95% (IC95%) por meio de modelos de regressão logística. Posteriormente, as variáveis que apresentaram valor de p ≤ 0,05 foram incluídas no modelo logístico para análise multivariada hierarquizada, conforme modelo teórico, e considerados como significantes valores de p ≤ 0,01 no modelo final após seleção por meio de *backward stepwise* . Os dados foram armazenados e analisados pelos programas SAS 9.4, SAS Inc, e SPSS *(Statistical Package for the Social Sciences) Statistics* ^®^ *26.0, IBM Inc.*

O estudo foi aprovado pelo Comitê de Ética do hospital (Protocolo 61/ 2017) e Plataforma Brasil (Parecer 160/2011).

## RESULTADOS

Foram identificados 1.132 registros elegíveis, sendo excluídos 102 pacientes internados no período em estudo por apresentarem dados incompletos no prontuário em relação às variáveis da assistência e 22 pacientes que evoluíram a óbito em menos de 48 horas de internamento. Dentre os 74 óbitos que permaneceram na análise (6,5% dos registros), 41 (55,4%) foram causados por IRAS e ocorreram entre três e 114 dias de vida.

Em relação ao desfecho, 427 registros (37,7%) tiveram IRAS tardia. A maioria dos recém-nascidos eram do sexo masculino (59,9%). A idade gestacional variou de 22 a 42 semanas, sendo que 62,5% eram prematuros, com predomínio de maiores ou iguais a 30 semanas e menores do que 34 semanas (37,5%). O peso ao nascer variou entre 415g e 6.200g, sendo que 60,7% eram de baixo peso (menos de 2.500g). Quanto ao índice de Apgar no 5º minuto de vida, dos valores válidos, 27% tinham índice menor do que sete.

Na análise bivariada das variáveis clínicas do recém-nascido observou-se um efeito protetor em relação a IRAS tardia em bebês do sexo feminino (OR = 0,71; IC95% 0,56–0,90) e em recém-nascido prematuro maior ou igual a 34 semanas e menor do que 37 semanas (OR = 0,48; IC95% 0,30–0,75). Quanto mais prematuro, maior é a associação com IRAS tardia, quase duas vezes maior no prematuro maior ou igual a 30 semanas e menor do que 34 semanas (OR = 1,93; IC95% 1,45–2,57) e cerca de dezoito vezes maior no prematuro menor do que 30 semanas (OR = 18,61; IC95% 9,84–35,22). O recém-nascido com peso ao nascer menor que 1.500g apresentou cerca de quatro vezes mais associação com IRAS tardia (OR = 4,18; IC95% 3,12–5,61), e o grupo dos menores que 2.500g apresentava um risco de cerca de duas vezes maior de IRAS tardia (OR = 2,27; IC95% 1,75–2,94). A asfixia associou-se a um risco cerca de três vezes maior de IRAS tardia (OR = 2,94; IC95% 2,17–3,84) ( Tabela 1 ). Foi observado que todas as variáveis apresentaram associação estatisticamente significativa a IRAS tardia (p < 0,0001), exceto sexo ( Tabela 1 ) e tempo de nutrição parenteral ( Tabela 2 ).

**Tabela 1:** Distribuição de casos e controles, com respectivos *odds ratio* , das variáveis relacionadas aos dados clínicos do recém-nascido. Ceará, Brasil, 2013 a 2017.

	IRAS tardia		OR (IC95%)	p
	Presente (n = 427) Caso	Ausente (n = 705) Controle		
Sexo	n (%)	n (%)		0,0065^b^
Feminino	193 (45,2%)	261 (37,0%)	0,71 (0,56–0,90)	
Masculino	234 (54,8%)	444 (63,0%)	1,0	
Classes de idade gestacional	n (%)	n (%)		< 0,0001^b^
≥ 37 semanas	122 (28,6%)	303 (43,0%)	1,0	
34 a 36 semanas	29 (6,8%)	151 (21,4%)	0,48 (0,30–0,75)	0,0012^a^
30 a 33 semanas	186 (43,6%)	239 (33,9%)	1,93 (1,45–2,57)	< 0,0001^a^
< 30 semanas	90 (21,1%)	12 (1,7%)	18,61 (9,84–35,22)	< 0,0001^a^
Peso de nascimento (g)				< 0,0001^a^
n	427	705		
Mediana (mín-máx)	1.405 (415–5.085)	2370 (550–6.200)		
Classes de peso de nascimento	n (%)	n (%)		< 0,0001^b^
≥ 2.500g	118 (27,6%)	327 (46,4%)	1,0	
Entre 1.500g e 2.499g	78 (18,3%)	225 (31,9%)	0,96 (0,69–1,34)	0,8131^a^
< 1.500g	231 (54,1%)	153 (21,7%)	4,18 (3,12–5,61)	< 0,0001^a^
Baixo peso	n (%)	n (%)		< 0,0001^b^
≥ 2.500g	118 (27,6%)	327 (46,4%)	1,0	
< 2.500g	309 (72,4%)	378 (53,6%)	2,27 (1,75–2,94)	< 0,0001^a^
Apgar do 5º minuto				< 0,0001^a^
n	385	652		
Mediana (mín-máx)	7,0 (0,0–10,0)	8,0 (1.0–10.0)		
Asfixia	n (%)	n (%)		< 0,0001^b^
Valores omissos	42	53		
Apgar ≥ 7	229 (59,5%)	528 (81,0%)	1,0	
Apgar < 7	156 (40,5%)	124 (19,0%)	2,94 (2,17–3,84)	< 0,0001^a^

^a^Kruskal-Wallis p-value.

^b^Chi-Square p-value.

**Tabela 2:** Distribuição de casos e controles, com respectivos *odds ratio* das variáveis relacionadas aos procedimentos utilizados durante o internamento e óbito. Ceará, Brasil, 2013 a 2017.

	IRAS tardia		OR (IC95%)	p
	Presente (n = 427) Casos	Ausente (n = 705) Controles		
Uso de ventilação mecânica	n (%)	n (%)		< 0,0001^b^
Não	76 (17,8%)	426 (60,4%)	1,0	
Sim	351 (82,2%)	279 (39,6%)	7,14 (5,26–9,09)	< 0,0001^a^
Tempo de ventilação (dias)			1,15 (1,11–1,20)	< 0,0001^a^
n	351	279		
Mediana (mín-máx)	9 (1–88)	4 (1–45)		
Uso de nutrição parenteral	n (%)	n (%)		< 0,0001^b^
Não	117 (27,4%)	484 (68,7%)	1,0	
Sim	310 (72,6%)	221 (31,3%)	5,88 (4,54–7,69)	< 0,0001^a^
Tempo de nutrição parenteral (dias)			1,08 (1,05–1,12)	0,0008^a^
n	310	221		
Mediana (mín-máx)	6 (1–59)	6 (1–27)		
Uso de cateter central	n (%)	n (%)		< 0,0001^b^
Não	21 (4,9%)	246 (34,9%)	1,0	
Sim	406 (95,1%)	459 (65,1%)	10,00 (6,66–16,66)	< 0,0001^a^
Tempo de cateter central (dias)			1,09 (1,07–1,11)	< 0,0001^a^
n	406	459		
Mediana (mín-máx)	10 (1–93)	7 (1–34)		
Número de cateteres			3,93 (3,02–5,12)	< 0,0001^a^
n	406	459		
Mediana (mín-máx)	2 (1–5)	1 (1–3)		
Realização de Cirurgia	n (%)	n (%)		< 0,0001^b^
Não	386 (90,4%)	687 (97,4%)	1,0	
Sim	41 (9,6%)	18 (2,6%)	4,00 (2,27–7,14)	< 0,0001^a^
Tempo total de internamento (dias)			1,06 (1,05–1,07)	< 0,0001^a^
n	427	705		
Mediana (mín-máx)	50 (3–198)	14 (2–100)		
Óbito	n (%)	n (%)		< 0,0001^b^
Não	368 (86,2%)	690 (97,9%)	1,0	
Sim	59 (13,8%)	15 (2,1%)	7,14 (4,16–12,50)	< 0,0001^a^

^a^Kruskal-Wallis p-value.

^b^Chi-Square p-value.

Na análise dos procedimentos, observou-se que 55,7% utilizaram ventilação mecânica e cerca de metade dos recém-nascidos permaneceram intubados até 5 dias, por um período que variou de um a 88 dias; 46,9% utilizaram nutrição parenteral; 76,4% utilizaram CVC, a maioria dos casos (62,2%) utilizou um cateter e 31,3%, dois cateteres; houve ainda 59 registros de cirurgia (5,2%). O tempo de internamento total (incluindo o período em unidade de risco intermediário), foi de até 21 dias para 50% dos recém-nascidos, com variação de dois a 198 dias ( Tabela 2 ).

Ao verificar as associações, observou-se que o uso de ventilação mecânica aumentou o risco de IRAS tardia em aproximadamente sete vezes (OR = 7,14; IC95% 5,26–9,09); cada dia de ventilação mecânica aumentou em 15% as chances de infecção (OR = 1,15; IC95% 1,11–1,20); o uso de nutrição parenteral, elevou quase seis vezes a chance (OR = 5,88; IC95% 4,54–7,69); bem como o tempo em dias de nutrição parenteral (OR = 1,08; IC95% 1,05–1,12). Além disso, o uso de CVC elevou a chance de infecção em dez vezes maior de IRAS tardia (OR = 10,00; IC95% 6,66–16,66) e cada dia de cateter aumentou em 9% essa chance (OR = 1,09; IC95% 1,07–1,11), e também identificou-se gradiente nessa associação, com o aumento do número de CVC utilizados, elevando a chance (OR = 3,93; IC95% 3,02–5,12). A realização de cirurgia, associou-se com chance quatro vezes maior de IRAS (OR = 4,00; IC95% 2,27–7,14); por fim, cada dia a mais de tempo total de internamento elevou em 6% as chances de IRAS tardia (OR = 1,06; IC95% 1,05–1,07) ( Tabela 2 ).

Dentre os casos encontrados, 164 tiveram dois ou mais episódios infecciosos assim distribuídos: 50 pacientes tiveram duas IRAS tardia, 20 tiveram três, 10 tiveram quatro, seis tiveram cinco, dois tiveram seis, e um teve sete IRAS tardia. Dentre os notificados como IRAS tardia, 54 (12,6%) apresentaram registro de hemocultura positiva para as infecções causadas por bactérias (n = 47), sete (14,9%) foram causadas por bactérias multirresistentes, as outras sete (13%) foram por fungos. As bactérias e/ou fungos identificados estão descritas na Tabela 3 .

**Tabela 3:** Resultados das hemoculturas positivas nas IRAS tardias em UTIN. Ceará, Brasil, 2013 a 2017.

Hemocultura	Frequência absoluta (n)	Frequência relativa (%)
Bactéria Gram-negativa	26	48,1
Bactéria Gram-positiva	21	38,9
Fungo	7	13,0
Total	54	100,0

A análise multivariada hierarquizada para o desfecho evidenciou associação significativa após ajuste no modelo final, como idade gestacional menor do que 30 semanas (OR = 5,62; IC95% 1,83–17,28); uso de ventilação mecânica (OR = 1,84; IC95% 1,26–2,68); uso de CVC (OR = 2,48; IC95% 1,40–4,37) e tempo de internação (OR = 1,06; IC95% 1,05–1,07) ( Tabela 4 ).

**Tabela 4:** Modelo final de acordo com as variáveis clínicas e dos procedimentos utilizados durante o internamento, com respectivos *odds ratio* , IC95%. Ceará, Brasil, 2013 a 2017.

	Odds ratio (IC95%)	p
Prematuro < 30 semanas	5,62 (1,83–17,28)	0,003^a^
Uso de ventilação mecânica	1,84 (1,26–2,68)	0,001^b^
Uso de cateter central	2,48 (1,40–4,37)	0,002^b^
Tempo total de internamento	1,06 (1,05–1,07)	< 0,0001^a^

^a^Covariate Wald p-value.

^b^Type 3 Wald p-value.

## DISCUSSÃO

Neste estudo, identificou-se que a ocorrência de IRAS tardia foi de 37,7%, e as chances de infecção aumentam conforme o nível de prematuridade, o uso de ventilação mecânica, o uso de CVC e o tempo de internamento.

A prevalência de IRAS tardia foi semelhante à de outros locais estudados: egípcio, 38,5%^20^, brasileiro, 34%^6^e indiano, 31%^21^, porém bem maior do que em Taiwan, 11,4%^22^e em estudo multicêntrico europeu, 10,7%^23^, esse desfecho esteve associado à prematuridade entre menores que 30 semanas de idade gestacional, uso de ventilação mecânica, uso de CVC e tempo de internamento.

Os agentes infecciosos predominantes neste estudo foram bactérias Gram-negativas (48,1%), Gram-positivas (38,9%) e fungos (13%), predomínio também observado em estudo indiano por bactérias Gram-negativas (61%) seguidos por Gram-positivas (39%)^24^; em estudo iraniano, por Gram-negativas (40%), Gram-positivas (20,7%) e fungos (6,7%)^25^, e na Jordânia, por Gram-negativas (62%), Gram-positivas (31%), e fungos (7%)^26^, onde também foi observado alta resistência bacteriana de cerca de 39%^27^, maior do que a observada neste estudo que foi de 14,9%.

Houve significativa associação entre IRAS e a idade gestacional nos mais prematuros, na análise bivariada, houve chance 18 vezes maior em menores de 30 semanas, permanecendo no modelo final após ajustes com outras variáveis. O recém-nascido já é naturalmente susceptível às infecções pela imaturidade de suas defesas imunológicas^3^, quando existem outros fatores como prematuridade e/ou baixo peso, o risco de complicações aumenta e, portanto, a necessidade de mais procedimentos invasivos e maior tempo de internamento. O prematuro apresenta frequentemente imaturidade pulmonar caracterizada como síndrome do desconforto respiratório (SDR) ou doença da membrana hialina, decorrente da deficiência do surfactante alveolar^28^. Devido à SDR, é comum o uso da ventilação mecânica para auxiliar os pulmões na manutenção das trocas gasosas^29^. Quanto mais prematuro, independente do peso, maior a gravidade da SDR e da necessidade de procedimentos invasivos, com aumento do risco de IRAS. Como frequentemente há relação direta entre peso mais baixo e prematuridade, essas variáveis podem se confundir nas análises estatísticas.

Neste estudo houve associação a IRAS tardia com o uso de CVC e de ventilação mecânica, o que ocorreu também em outros estudos, embora com valores maiores: uso de CVC (OR = 4,32; IC95% 1,95–9,56) e uso de ventilação mecânica (OR = 3,42; IC95% 2,17–5,41)^6^. Em estudo transversal realizado entre 2013 e 2015, no Rio Grande do Sul, o uso de ventilação mecânica e realização de cirurgia apresentaram associação estatística significativa para IRAS em neonatos com hemocultura positiva (47,97% dos infectados)^10^; outro trabalho em Londrina, no Paraná, no período de 2001 a 2005, observou associação relevante com intubação e uso de CVC^21^.

Em metanálise de 22 estudos (2.270 recém-nascidos com infecção e 21.605 sem infecção), publicada em 2019^30^, foram associados como risco para IRAS tardia: peso < 2.500g (RR = 3,44; IC95% 2,31–5,11), prematuridade (RR = 3,85; IC95% 1,87–7,92), uso de ventilação mecânica (RR = 3,16; IC95% 2,21–4,50), e asfixia (RR = 1,68; IC95% 1,04–2,71). Este trabalho também associou IRAS a prematuridade e ventilação mecânica. Em estudo indiano, houve associação com prematuridade (OR = 3,05; IC95% 1,94–9,88), CVC (OR = 15,11; IC95% 3,40–67,01), ventilação mecânica (OR = 8,94; IC95% 1,32–60,31) e tempo de permanência em UTIN maior do que 10 dias (OR = 4,09; IC95% 1,05–16,70)^25^, o que também ocorreu neste estudo, embora com valores menores. A utilização de cateteres intravasculares tornou-se nos últimos anos uma prática quase indispensável em cuidados intensivos, por ser um acesso vascular de alto débito^18^, além da dificuldade de acesso vascular periférico em recém-nascidos. A desvantagem do acesso central é o aumento do risco de infecção local ou sistêmica, podendo ocasionar infecção da corrente sanguínea associada ao CVC^18^. Como frequentemente o recém-nascido faz uso de mais de um acesso central, maior é o manuseio e maior o risco de IRAS^18^. Quanto maior o tempo de internação e mais procedimentos invasivos forem necessários para assistência ao recém-nascido, com o uso de dispositivos invasivos como cateteres centrais e ventilação mecânica, por exemplo, maior o manuseio e risco de IRAS.

Apesar dos resultados encontrados neste estudo serem consistentes com a literatura nacional e internacional, existem limitações dignas de nota. O uso de dados secundários retrospectivamente pode levar a registros incompletos no prontuário, principalmente em relação aos dados do recém-nascido e ao tempo de uso dos procedimentos invasivos. Isso foi contornado através de revisão de todo o internamento por meio dos registros de declaração de nascido vivo e/ou de relatório de transferência inter-hospitalar e das evoluções diárias de toda a equipe multiprofissional.

Foi evidenciado que a IRAS tardia ainda é muito frequente em UTIN, que os prematuros ainda são um grupo de risco bastante vulnerável à infecção e que os procedimentos invasivos, essenciais para a maioria dos neonatos criticamente doentes, devem ser criteriosamente indicados.

## CONCLUSÕES

Concluímos que, embora não seja possível evitar procedimentos invasivos em neonatos críticos, as práticas de inserção, instalação e manutenção desses dispositivos devem ser rigorosamente revisadas junto às comissões de controle de infecção hospitalar e de segurança na assistência, obedecendo a protocolos atualizados e vigilantes. O uso criterioso de antimicrobianos, aplicados para evitar o aparecimento de bactérias multirresistentes também é ação prioritária. Para a prematuridade, a melhor estratégia é prevenir a ocorrência de partos prematuros, pois o meio intrauterino na maioria das vezes, com o seguimento pré-natal adequado, é o mais seguro para a permanência do feto o maior tempo possível, até próximo ou atingir a idade gestacional de termo.
